# Genetic variants associated with diseases in Afghan population

**DOI:** 10.1002/mgg3.1608

**Published:** 2021-01-24

**Authors:** Suleman Khan Zadran, Muhammad Ilyas, Shamsia Dawari

**Affiliations:** ^1^ Department of Pharmacy and Biotechnology University of Bologna Bologna Italy; ^2^ Centre for Omic Sciences Islamia College University Peshawar Peshawar Pakistan; ^3^ Milli Institute of Higher Education Kabul Afghanistan

Afghanistan is a landlocked country, held a strategic position in Central Asia maintains its importance with approximately 32.9 million in 2017 (NISA, 2019). Over the years, instability in Afghanistan due to ongoing wars and regional issues has been affecting almost every system of the country like education and health. Particularly, higher education systems have been badly affected over the years. Science and technology sector in the country have never been addressed properly instead most of the work in this area has been done by the international organizations.

In Afghanistan, the prevalence of cousin marriages is estimated to be 46.2%. The prevalent type of cousin marriage is first cousin marriage (27.8%), followed by double first cousin marriage (6.9%), second cousin (5.8%), and third cousin (3.9%) (Saify & Saadat, 2012). Such marriages became the main reason to get genetically disabled children. International research groups working on genetic characterization of inherited diseases focus on such large consanguineous families with genetic disorders. In the current article, we tried to compile all the disease‐associated mutations reported in Afghanistan human populations which can be further used for developing personalized medicine.

Extensive literature search related to genetic disorders/problems was carried out, comprising of all published articles from 1973 to 2019. Various databases were searched for collecting relevant literature like PubMed and Google Scholars using different key words.

Hereditary disorder might be one of the fundamental causes of the high death rate in Afghanistan. Based on this study, infants under the age of 2 years are mostly experiencing metabolic disorders and its frequency is up to 38.9%, followed by children in the age group 3–11 years (22.2%). Adolescents have comparatively less percentage (12.5%), but with diverse genetic anomalies, and adults have a high percentage (25.0%) of various genetic disorders, while older people (1.4%) are only affected by neurological disorders as shown in Figure 1. Metabolic disorders represent the most prevalent anomalies in the Afghan population, and until now, 31 different kinds of metabolic disorders have been reported. Previous reports in the literature often cite an accumulated incidence that about 2%–3% of the world population is suffering from inborn metabolism errors (Wasim et al., 2018). It is estimated that worldwide there have been prevailing inherited metabolism disorders of 1 in 784 births and around 1000 inborn metabolism errors are identified to date (Sanderson et al., 2006). Risk factors that contribute singly or interactively to birth defects comprise genetics, consanguineous marriage, ongoing warfare, and air pollution, synthetic compounds, physical and natural issues, and maternal elements (Risch et al., 2002; Wani et al., 2015). No genetic diagnostic laboratory for screening in Afghanistan is available to date. The percentage may be higher as shown in the graph because no studies have been conducted concerning the prevalence of genetic anomalies and screening.

**FIGURE 1:**
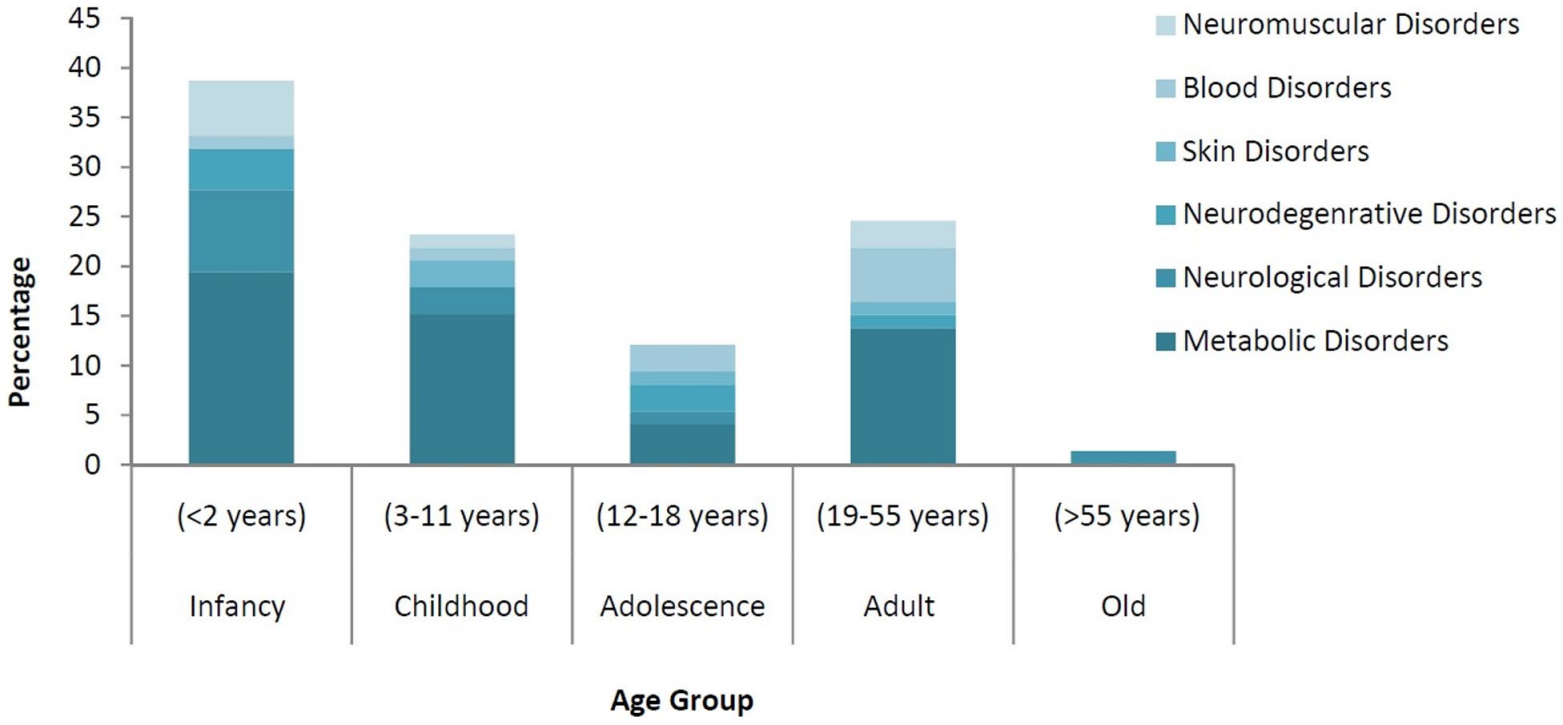
Representation of genetic disorders in different age group of Afghan population: Infant = 38.9%; Childhood = 22.2%, Adolescence = 12.5%; Adult = 25.0%; and Old = 1.4%

It is further observed that genetic disorders are prevalent in both sexual orientations among the Afghan population. However, males are most affected (50%) than females (44%). According to previous studies, men are more vulnerable than women to genetic disorders (Kraemer, 2000; Nugentet al., 2018).

Mutations (SNPs and Indels) whether inherited or acquired are attributed to most of the associated genetic disorders as discussed previously (Feuk et al., 2006). In the Afghan population, the wide range of variations is reported on all chromosomes except chromosomes 4, 19, and Y. Based on the current data, chromosome number 6 carried the highest mutations rate than others as shown in Figure 2. Chromosome numbers 11 and 12 also carry slightly high mutations frequency, while the rest of all carried lesser but diverse mutations. Chromosomes 7, 8, 9, 13, and 17 carry single mutations that are not specified according to the types of mutations (Table S1).

**FIGURE 2:**
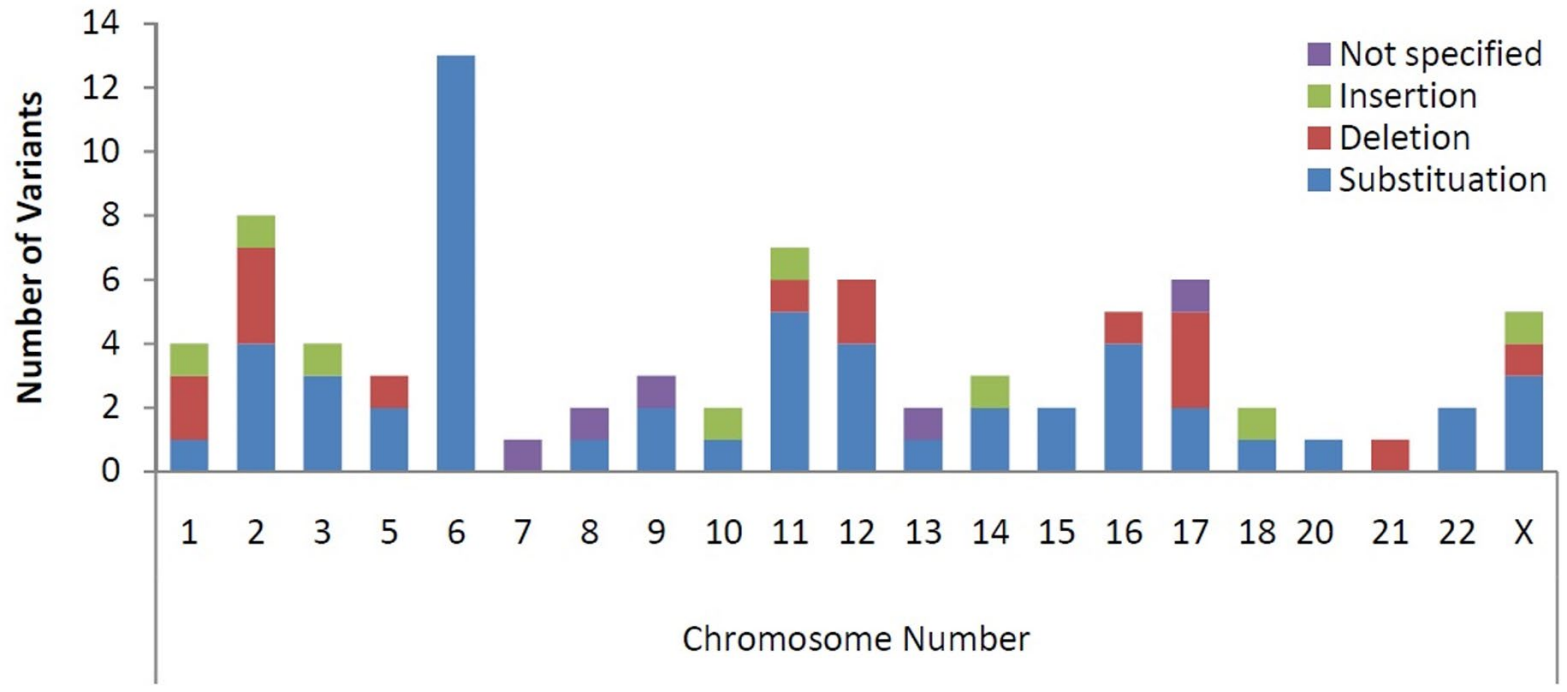
Representation of variants on each chromosome and mutation types

The pattern of inheritance of genetic abnormalities in Afghanistan has a significant impact on prevalence. Autosomal recessive genetic abnormalities were observed at most and 75.4% of the total reported cases, followed by autosomal dominant 19.7% (Figure 3). The main reason behind the high percentage of autosomal recessive conditions is the union between groups of people known to share genetic traits inherited from one or more common ancestors. Afghanistan has the highest rate of consanguinity among Asian countries (Saadat & Zarghami, 2018; Saify & Saadat, 2012).

**FIGURE 3:**
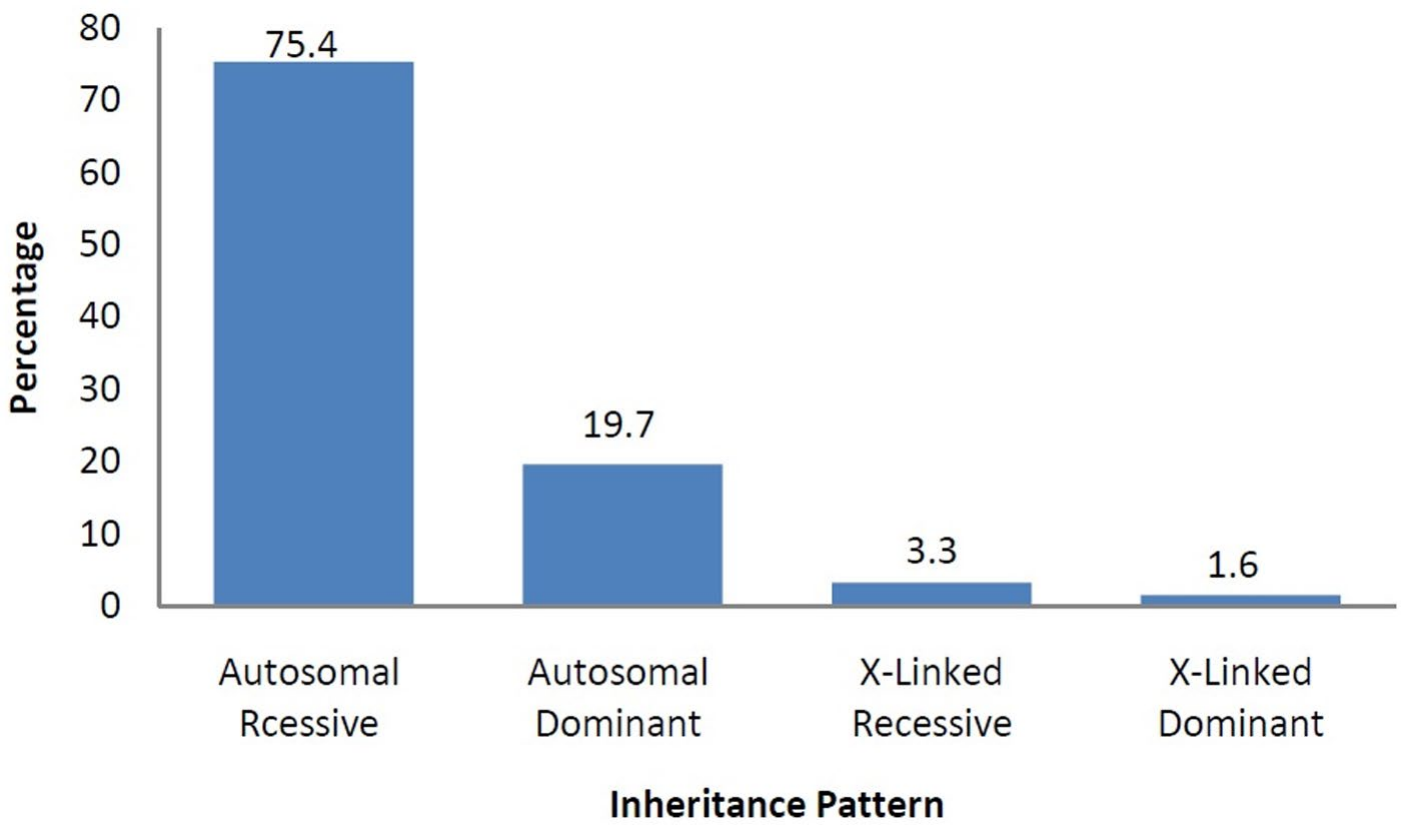
Representation of inheritance pattern of genetic disorders in the Afghan population, 75.4% of cases are reported as autosomal recessive conditions, 19.7% autosomal dominant. X‐linked recessive 3.3%, while the lowest percentage 1.6 was reported on X‐linked dominant conditions

We have provided an overview of genetic disorders reported in the Afghan population. The prevalence of genetic disorders is increasing year by year as a result of high consanguinity, the ongoing war for four decades, and chronic exposure to various emissions in the environment. The percentage of genetic disorders increases briskly if such circumstances persist. Awareness about the negative effects of consanguinity and the dangerous effects of various pollutants on the incoming progeny should be generated in such communities. The government must establish diagnostic and rehabilitation centers for reducing the impacts of genetic disorders. Risk families are advised to carry out early detection, diagnosis, and intervention to prevent death or disability, and allow children to grow normally. The Afghan population is in dire need of health attention.

## 1 COMPLIANCE WITH ETHICAL STANDARDS

This article does not contain any studies with human or animal subjects performed by any of the authors.

## CONFLICT OF INTEREST

The authors declare that they have no conflict of interest.

## AUTHOR CONTRIBUTIONS

SKZ and MI have conceived, designed, and directed the study; SKZ and SD collected the data. All the authors approved the final manuscript and contributed critical revisions to its intellectual content.

## Supporting information

Table S1

## Data Availability

Data sharing not applicable to this article as no datasets were generated or analyzed during the current study.
